# Supplementary material for: Combined transcriptome and proteome profiling reveal cell-type-specific functions of Drosophila garland and pericardial nephrocytes
Source: Commun Biol. 2024 Nov 1;7:1424. doi: 10.1038/s42003-024-07062-z (PMC11530456; doi:10.1038/s42003-024-07062-z)
Supplement: Supplementary file 2 — Supplementary information [file 42003_2024_7062_MOESM2_ESM.pdf]

Supporting Information captions:

Supplementary Figures

Fig. S1

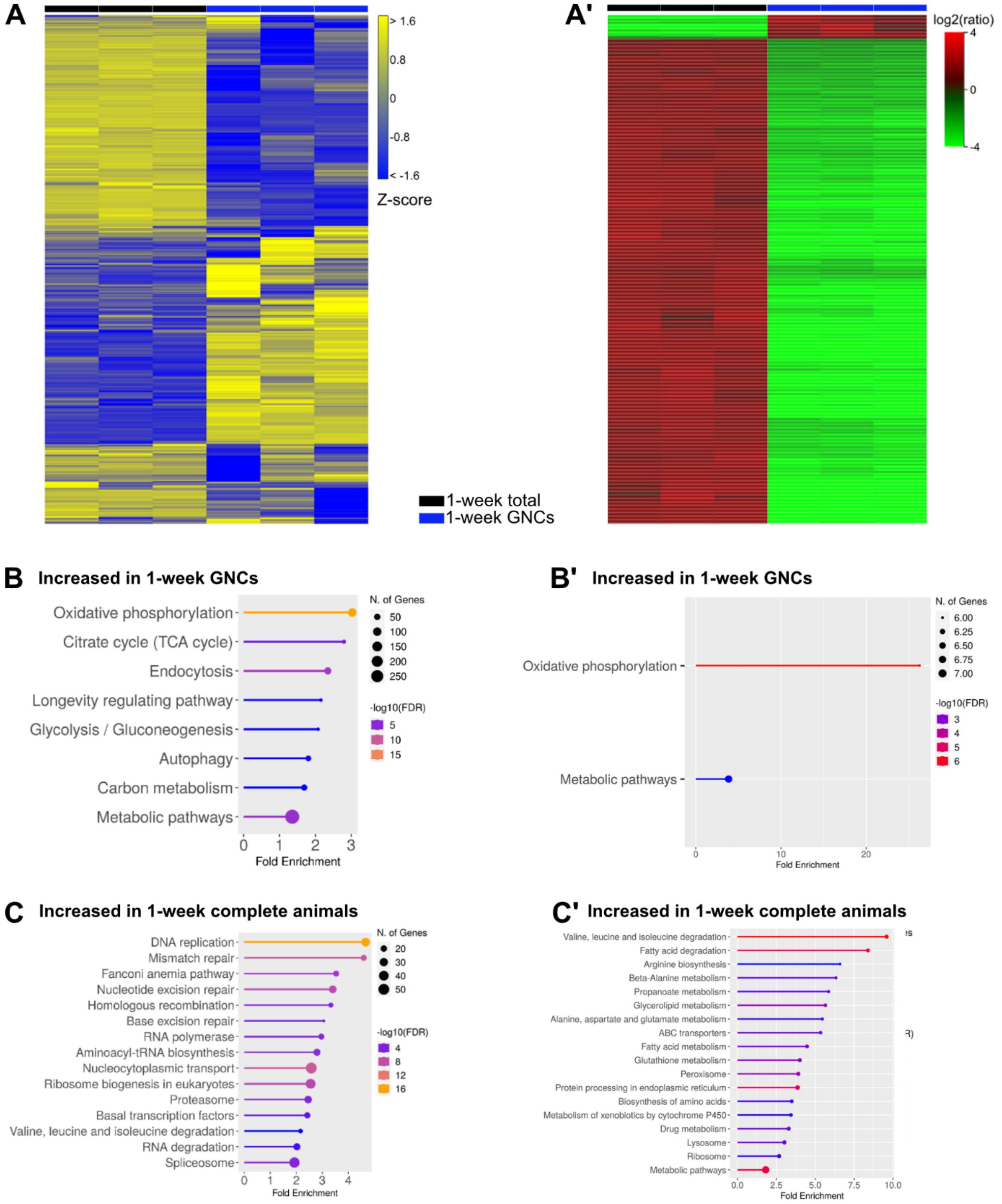

Figure S1: Core characteristics of garland nephrocytes at the protein and transcript level. (A) Heatmap of RNAseq replicates (minimum fold change 1.5, FDR p-value cutoff 0.05) from 1-week-old

complete animals (black bar) and 1-week-old GNCs (blue bar). (A´) Heatmap of proteome replicates isolated from 1-week-old complete animals (black bar) and 1-week-old GNCs (blue bar). (B) KEGG pathways (transcriptome-based) enriched in 1-week-old GNCs, relative to 1-week-old total animals. (B´) KEGG pathways (proteome-based) enriched in 1-week-old GNCs, relative to 1-week-old total animals. (C) KEGG pathways (transcriptome-based) enriched in 1-week-old total animals, relative to 1-week-old GNCs. (C´) KEGG pathways (proteome-based) enriched in 1-week-old total animals, relative to 1-week-old GNCs.

Fig. S2

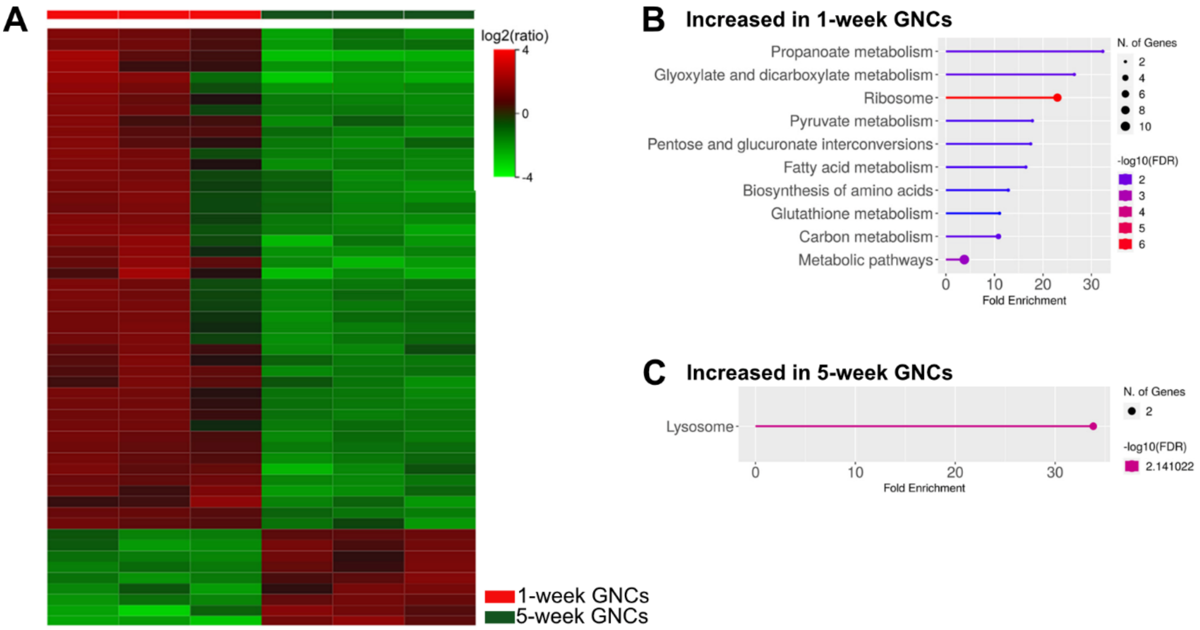

Figure S2: Proteomic signatures indicate similar cellular functions of 1-week-old and 5-week-old garland nephrocytes. (A) Heatmap of proteome replicates isolated from 1-week-old GNCs (red bar) and 5-week-old GNCs (green bar). (B) KEGG pathways enriched in 1-week-old GNCs, relative to 5-week-old GNCs. (C) KEGG pathways enriched in 5-week-old GNCs, relative to 1-week-old GNCs.

Fig. S3

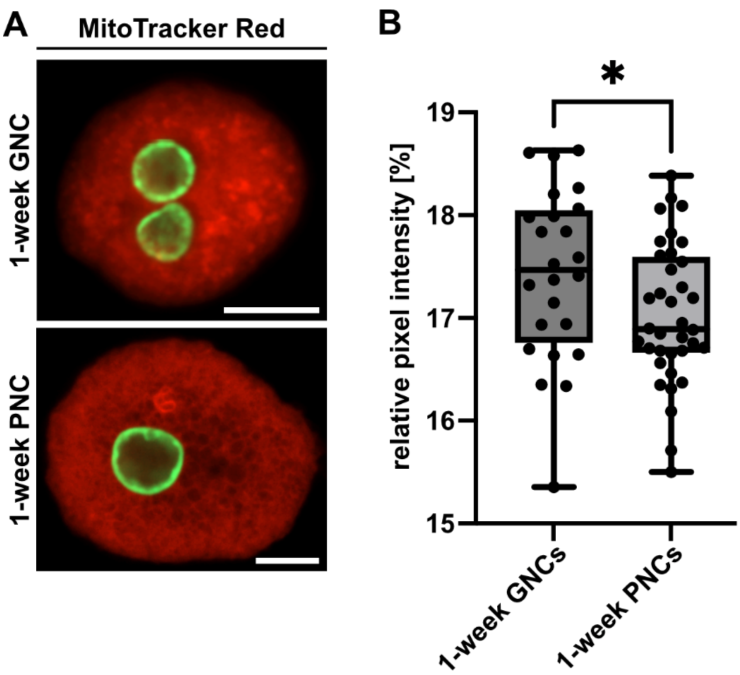

Figure S3: MitoTracker staining in nephrocytes. (A) Representative images of garland nephrocytes (GNCs) and pericardial nephrocytes (PNCs) isolated from *hand*C-GFP expressing animals (green) and stained with MitoTracker (red). Scale bars: 10 µm. (B) Quantification of the respective pixel intensity values reveals a significantly increased abundance of mitochondria in GNCs relative to PNCs. For the boxplots, the centre line indicates the median, the upper and lower bounds indicate the 75th and 25th percentiles, respectively, and the whiskers indicate the minimum and maximum. For each analysis, a minimum of 20 individual cells was measured. Asterisks indicate statistically significant differences between the two cell types (*$p < 0.05$, two-tailed, unpaired t-test).

Fig. S4

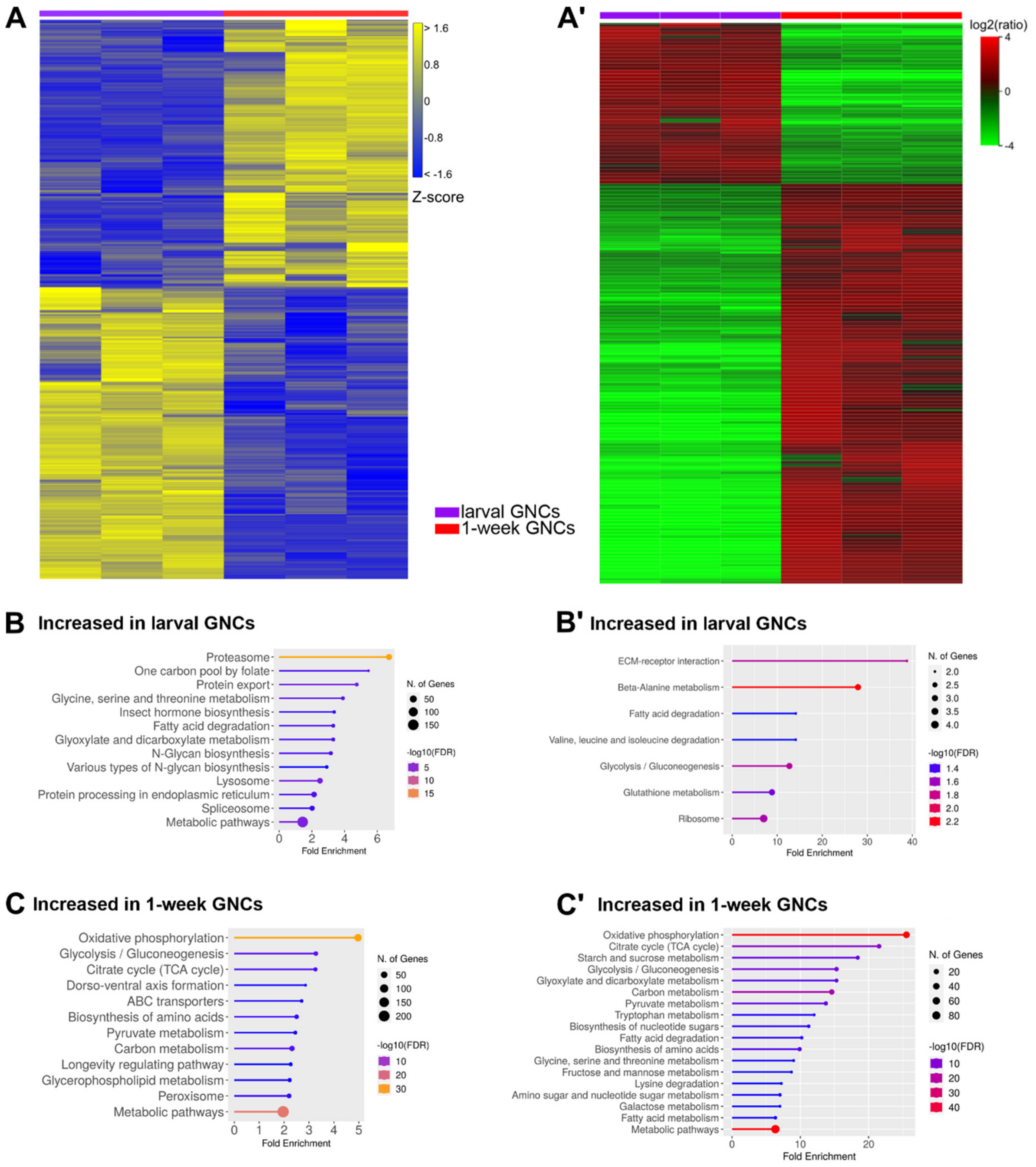

Figure S4: Garland nephrocytes exhibit developmental stage-specific transcriptomic and proteomic signatures. (A) Heatmap of RNAseq replicates (minimum fold change 1.5, FDR p-value cutoff 0.05) from 3rd instar larval GNCs (purple bar) and 1-week-old adult GNCs (red bar). (A´) Heatmap of proteome replicates from 3rd instar larval GNCs (purple bar) and 1-week-old adult GNCs (red bar). (B) KEGG pathways (transcriptome-based) enriched in 3rd instar larval GNCs, relative to 1-week-old adult GNCs. (B´) KEGG pathways (proteome-based) enriched in 3rd instar larval GNCs, relative to 1-week-old adult GNCs. (C) KEGG pathways (transcriptome-based) enriched in 1-week-old adult GNCs, relative to 3rd instar larval GNCs. (C´) KEGG pathways (proteome-based) enriched in 1-week-old adult GNCs, relative to 3rd instar larval GNCs.

Fig. S5

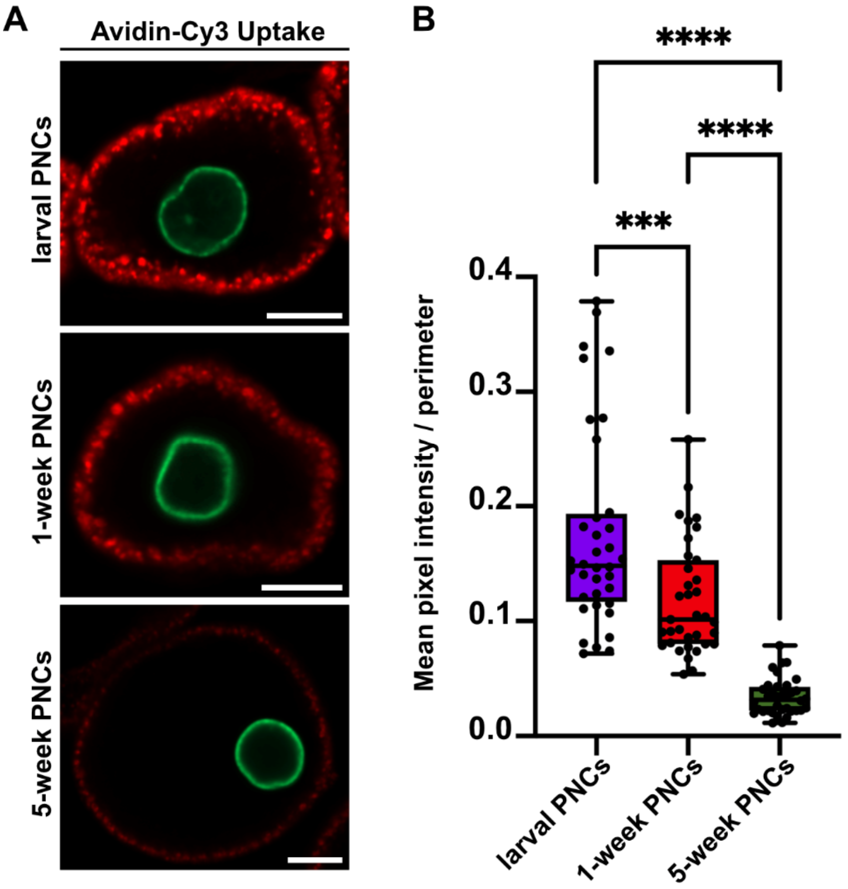

Figure S5: Endocytic activity in nephrocytes decreases with age. (A) Representative images of pericardial nephrocytes (PNCs) of different developmental stages and ages isolated from *hand*C-GFP expressing animals (green) and incubated with Cy3-labelled avidin (red). Scale bars: 10 µm. (B) Endocytic uptake efficiency was determined by pixel intensity measurements. Signal intensities were normalised to the individual cell perimeters. For all boxplots, the centre line indicates the median, the upper and lower bounds indicate the 75th and 25th percentiles, respectively, and the whiskers indicate the minimum and maximum. For each analysis, a minimum of 30 individual cells was measured. Asterisks indicate statistically significant differences between the two cell types (***$p < 0.0005$, ****$p < 0.0001$, one-way ANOVA).

Fig. S6

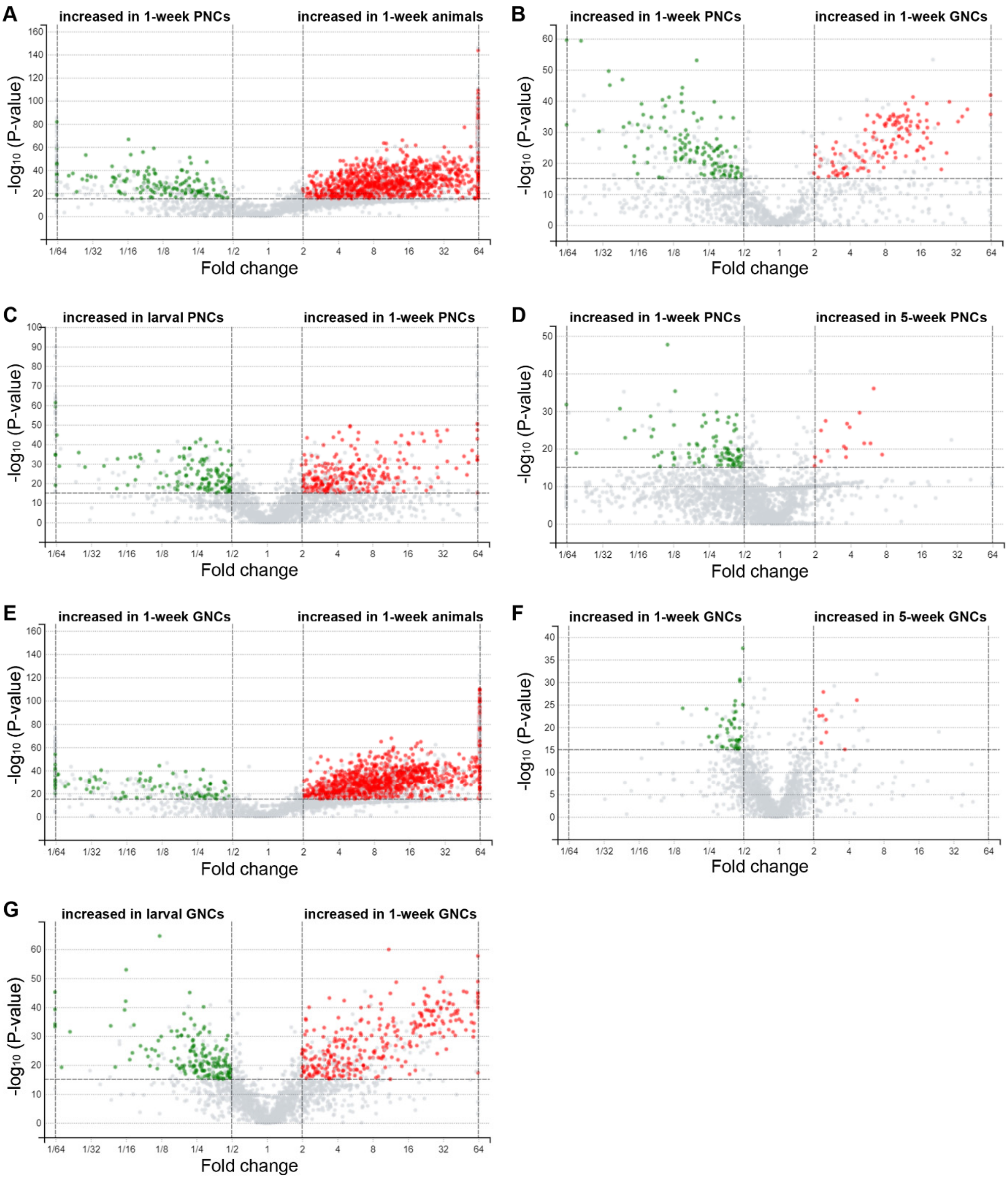

Figure S6: Volcano plots for all proteome comparisons performed in this study. (A) 1-week PNCs vs 1-week total animals; (B) 1-week PNCs vs 1-week GNCs; (C) 3rd instar larval PNCs vs 1-week PNCs; (D) 1-week PNCs vs 5-week PNCs; (E) 1-week GNCs vs 1-week total animals; (F) 1-week GNCs vs 5-week GNCs; (G) 3rd instar larval GNCs vs 1-week GNCs.
