# Supplementary material for: Combined transcriptome and proteome profiling reveal cell-type-specific functions of Drosophila garland and pericardial nephrocytes
Source: Commun Biol. 2024 Nov 1;7:1424. doi: 10.1038/s42003-024-07062-z (PMC11530456; doi:10.1038/s42003-024-07062-z)
Supplement: Supplementary file 3 — Description of Additional Supplementary Files [file 42003_2024_7062_MOESM3_ESM.pdf]

Description of Additional Supplementary Files

Supplementary Data 1a: 1w Total vs 1w PNCs (Figure 2F)
Depicted are proteins that exhibit altered amounts in 1w complete animals, relative to 1w pericardial cells.

Supplementary Data 1b: Increased in 1w PNCs vs Total (Figure 2G)
Depicted are proteins with increased abundance in 1w pericardial cells, relative to 1w complete animals. Proteins are allocated to their individual KEGG pathways.

Supplementary Data 1c: Increased in 1w Total vs PNCs (Figure 2H)
Depicted are proteins with increased abundance in 1w complete animals, relative to 1w pericardial cells. Proteins are allocated to their individual KEGG pathways.

Supplementary Data 2a: 1w PNCs vs 1w GNCs (Figure 5A')
Depicted are proteins that exhibit altered amounts in 1w pericardial cells, relative to 1w garland cells.

Supplementary Data 2b: Increased in 1w PNCs vs GNCs (Figure 5B')
Depicted are proteins with increased abundance in 1w pericardial cells, relative to 1w garland cells. Proteins are allocated to their individual KEGG pathways.

Supplementary Data 2c: Increased in 1w GNCs vs PNCs (Figure 6A')
Depicted are proteins with increased abundance in 1w garland cells, relative to 1w pericardial cells. Proteins are allocated to their individual KEGG pathways.

Supplementary Data 3a: 1w Total vs 1w GNCs (Figure S1A')
Depicted are proteins that exhibit altered amounts in 1w complete animals, relative to 1w garland cells.

Supplementary Data 3b: Increased in 1w GNCs vs Total (Figure S1B')
Depicted are proteins with increased abundance in 1w garland cells, relative to 1w complete animals. Proteins are allocated to their individual KEGG pathways.

Supplementary Data 3c: Increased in 1w Total vs 1w GNCs (Figure S1C')
Depicted are proteins with increased abundance in 1w complete animals, relative to 1w garland cells. Proteins are allocated to their individual KEGG pathways.

Supplementary Data 4a: larval PNCs vs 1w PNCs (Figure 7A')
Depicted are proteins that exhibit altered amounts in larval pericardial cells, relative to 1w pericardial cells.

Supplementary Data 4b: Increased in larval PNCs vs 1w PNCs (Figure 7B')
Depicted are proteins with increased abundance in larval pericardial cells, relative to 1w pericardial cells. Proteins are allocated to their individual KEGG pathways.

Supplementary Data 4c: Increased in 1w PNCs vs larval PNCs (Figure 7C')

Depicted are proteins with increased abundance in 1w pericardial cells, relative to larval pericardial cells. Proteins are allocated to their individual KEGG pathways.

Supplementary Data 5a: 1w PNCs vs 3w PNCs (Figure 8G)
Depicted are proteins that exhibit altered amounts in 1w pericardial cells, relative to 3w pericardial cells.

Supplementary Data 5b: 1w PNCs vs 5w PNCs (Figure 8G)
Depicted are proteins that exhibit altered amounts in 1w pericardial cells, relative to 5w pericardial cells.

Supplementary Data 5c: Increased in 3w PNCs vs 1w PNCs (Figure 8H)
Depicted are proteins with increased abundance in 3w pericardial cells, relative to 1w pericardial cells. Proteins are allocated to their individual KEGG pathways.

Supplementary Data 5d: Reduced in 5w PNCs vs 1w PNCs (Figure 8I)
Depicted are proteins with reduced abundance in 5w pericardial cells, relative to 1w pericardial cells. Proteins are allocated to their individual KEGG pathways.

Supplementary Data 6a: 1w GNCs vs 5w GNCs (Figure S2A)
Depicted are proteins that exhibit altered amounts in 1w garland cells, relative to 5w garland cells.

Supplementary Data 6b: Increased in 1w GNCs vs 5w GNCs (Figure S2B)
Depicted are proteins with increased abundance in 1w garland cells, relative to 5w garland cells. Proteins are allocated to their individual KEGG pathways.

Supplementary Data 6c: Increased in 5w GNCs vs 1w GNCs (Figure S2C)
Depicted are proteins with increased abundance in 5w garland cells, relative to 1w garland cells. Proteins are allocated to their individual KEGG pathways.
