# Supplementary material for: Combined transcriptome and proteome profiling reveal cell-type-specific functions of Drosophila garland and pericardial nephrocytes
Source: Commun Biol. 2024 Nov 1;7:1424. doi: 10.1038/s42003-024-07062-z (PMC11530456; doi:10.1038/s42003-024-07062-z)
Supplement: Supplementary file 5 — Reporting Summary [file 42003_2024_7062_MOESM5_ESM.pdf]

nature research

Corresponding author(s): Achim Paululat
Last updated by author(s): Feb 7, 2024

Reporting Summary

Nature Research wishes to improve the reproducibility of the work that we publish. This form provides structure for consistency and transparency in reporting. For further information on Nature Research policies, see our Editorial Policies and the Editorial Policy Checklist.

Statistics

For all statistical analyses, confirm that the following items are present in the figure legend, table legend, main text, or Methods section.

n/a	Confirmed	
☐	☒	The exact sample size (*n*) for each experimental group/condition, given as a discrete number and unit of measurement
☐	☒	A statement on whether measurements were taken from distinct samples or whether the same sample was measured repeatedly
☐	☒	The statistical test(s) used AND whether they are one- or two-sided<br>*Only common tests should be described solely by name; describe more complex techniques in the Methods section.*
☒	☐	A description of all covariates tested
☐	☒	A description of any assumptions or corrections, such as tests of normality and adjustment for multiple comparisons
☐	☒	A full description of the statistical parameters including central tendency (e.g. means) or other basic estimates (e.g. regression coefficient) AND variation (e.g. standard deviation) or associated estimates of uncertainty (e.g. confidence intervals)
☐	☒	For null hypothesis testing, the test statistic (e.g. *F*, *t*, *r*) with confidence intervals, effect sizes, degrees of freedom and *P* value noted<br>*Give P values as exact values whenever suitable.*
☒	☐	For Bayesian analysis, information on the choice of priors and Markov chain Monte Carlo settings
☒	☐	For hierarchical and complex designs, identification of the appropriate level for tests and full reporting of outcomes
☒	☐	Estimates of effect sizes (e.g. Cohen's *d*, Pearson's *r*), indicating how they were calculated

Our web collection on statistics for biologists contains articles on many of the points above.

Software and code

Policy information about availability of computer code

Data collection	Zen (version 2.6, blue edition, Zeiss)
Data analysis	ImageJ (version 1.52n, Schneider et al., 2012, PMID: 22930834)<br>PEAKS Studio software (version 10.6, Bioinformatics Solutions)<br>CLC Genomics Workbench software (Qiagen)<br>GraphPad Prism (version 5.03, GraphPad Software)<br>ShinyGO (v0.77, RRID:SCR_019213)<br>Affinity Designer (version 1.8.5.703, Serif)

For manuscripts utilizing custom algorithms or software that are central to the research but not yet described in published literature, software must be made available to editors and reviewers. We strongly encourage code deposition in a community repository (e.g. GitHub). See the Nature Research guidelines for submitting code & software for further information.

Data

Policy information about availability of data

All manuscripts must include a data availability statement. This statement should provide the following information, where applicable:

- Accession codes, unique identifiers, or web links for publicly available datasets
- A list of figures that have associated raw data
- A description of any restrictions on data availability

The data supporting the findings from this study are available within the manuscript and its supplementary information. The proteomics data have been deposited in the ProteomeXchange Consortium via the PRIDE partner repository with the dataset identifier PXD047262 (https://www.ebi.ac.uk/pride/archive/projects/PXD047262; Reviewer login: reviewer_pxd047262@ebi.ac.uk, Password: glSs7p8p). SwissProt database (UP000000803, www.uniprot.org/proteomes/

UP000000803) was used to determine peptide-specific amino acid sequences. Transcriptomic datasets have been deposited in the GEO database: GSE250029 (Reviewer token: qdcrgigwftgphgj). Source data are provided with this paper.

Field-specific reporting

Please select the one below that is the best fit for your research. If you are not sure, read the appropriate sections before making your selection.

☒ Life sciences ☐ Behavioural & social sciences ☐ Ecological, evolutionary & environmental sciences

For a reference copy of the document with all sections, see nature.com/documents/nr-reporting-summary-flat.pdf

Life sciences study design

All studies must disclose on these points even when the disclosure is negative.

Sample size	Sample sizes were chosen based on established protocols and previous publications. Please see: Bossen et al., 2023 (PMID: 36982710), Ivy et al., 2015 (PMID: 26301956), Zeng et al., 2015 (PMID: 25596379), Hartley et al., 2016 (PMID: 26839388)
Data exclusions	For mass spectrometry analyses, only proteins with quantification being based on two or more detected peptides were considered. Proteins with only one detected peptide were excluded from further analysis.
Replication	To ensure reproducibility, all generated datasets were based on at least three individual biological replicates. All attempts at replication were successful.
Randomization	No randomization was necessary for this study because investigators were comparing proteomic / transcriptomic samples under well controlled conditions (e.g. variable age or developmental stage). No human subjects were used in the study. Randomization is not generally used in this field.
Blinding	Investigators were not blind to the group assignment, as the experimental design prescribed specific age or developmental stages for the individual groups. However, all raw data generated were re-analyzed by at least one additional person on a sample basis. Blinding is not typically used in this field.

Reporting for specific materials, systems and methods

We require information from authors about some types of materials, experimental systems and methods used in many studies. Here, indicate whether each material, system or method listed is relevant to your study. If you are not sure if a list item applies to your research, read the appropriate section before selecting a response.

Materials & experimental systems

n/a	Involved in the study	
☒	☐	Antibodies
☒	☐	Eukaryotic cell lines
☒	☐	Palaeontology and archaeology
☐	☒	Animals and other organisms
☒	☐	Human research participants
☒	☐	Clinical data
☒	☐	Dual use research of concern

Methods

n/a	Involved in the study	
☒	☐	ChIP-seq
☒	☐	Flow cytometry
☒	☐	MRI-based neuroimaging

Animals and other organisms

Policy information about studies involving animals; ARRIVE guidelines recommended for reporting animal research

Laboratory animals	Drosophila melanogaster lines used:<br><br>w1118 (RRID:BDSC_5905)<br><br>handC-GFP (Sellin et al., 2006, PMID: 16455308)<br><br>handC-mCherry (Paululat and Heinisch, 2012, PMID: 23026211)<br><br>Klf15NN (Ivy et al., 2015, PMID: 26301956)<br><br>All antimicrobial peptide reporter lines used are described in (Tzou et al., 2000, PMID: 11114385)
Wild animals	This study did not involve wild animals.

Field-collected samples	This study did not involve samples collected from the field.
Ethics oversight	The Drosophila work performed in this study did not require any ethical approval or guidance.

Note that full information on the approval of the study protocol must also be provided in the manuscript.
